# Primary intracerebral osteosarcoma: a rare case report and review

**DOI:** 10.1186/s40064-016-3678-z

**Published:** 2016-11-22

**Authors:** Song-Ping Chen, Jin-Long Tang, Xiu-Liang Zhu

**Affiliations:** 1 Department of Radiology, The People’s Hospital of Zhenhai, No. 718 Naner West Road, Ningbo, 315202 Zhejiang China; 2 Department of Pathology, The Second Affiliated Hospital, Zhejiang University School of Medicine, No. 88 Jiefang Road, Hangzhou, 310009 Zhejiang China; 3 Department of Radiology, The Second Affiliated Hospital, Zhejiang University School of Medicine, No. 88 Jiefang Road, Hangzhou, 310009 Zhejiang China

**Keywords:** Osteosarcoma, Brain, Primary, MRI

## Abstract

**Background:**

Primary intracranial osteosarcoma is a extremely rare disease entity. We describe a case of primary intracerebral osteosarcoma in an adult brain.

**Case description:**

A patient who presented with a 1-week history of headaches, and MRI examination was performed. The immunohistochemical diagnosis confirmed primary intracerebral osteosarcoma. The patient was treated with a surgical resection of the tumor.

**Conclusion:**

Primary osteosarcomas occurring in the brain are extremely rare. The MRI images did not provide a specific pretreatment diagnosis, and the histopathology was the mainstay in establishing the diagnosis.

## Background

Osteosarcoma is a highly malignant neoplasm of bone and can occur anywhere in the body, but is most commonly found in the long bones. Extraskeletal osteosarcomas occurring in tissues other than bone, are rare. Primary intracranial osteosarcomas are extremely rare, developing from leptomeningeal sheats around blood vessels or from vessel walls themselves (Ashkan et al. 1998). Here we report a case of primary intracerebral osteosarcoma.

## Case report

**Fig. 1:**
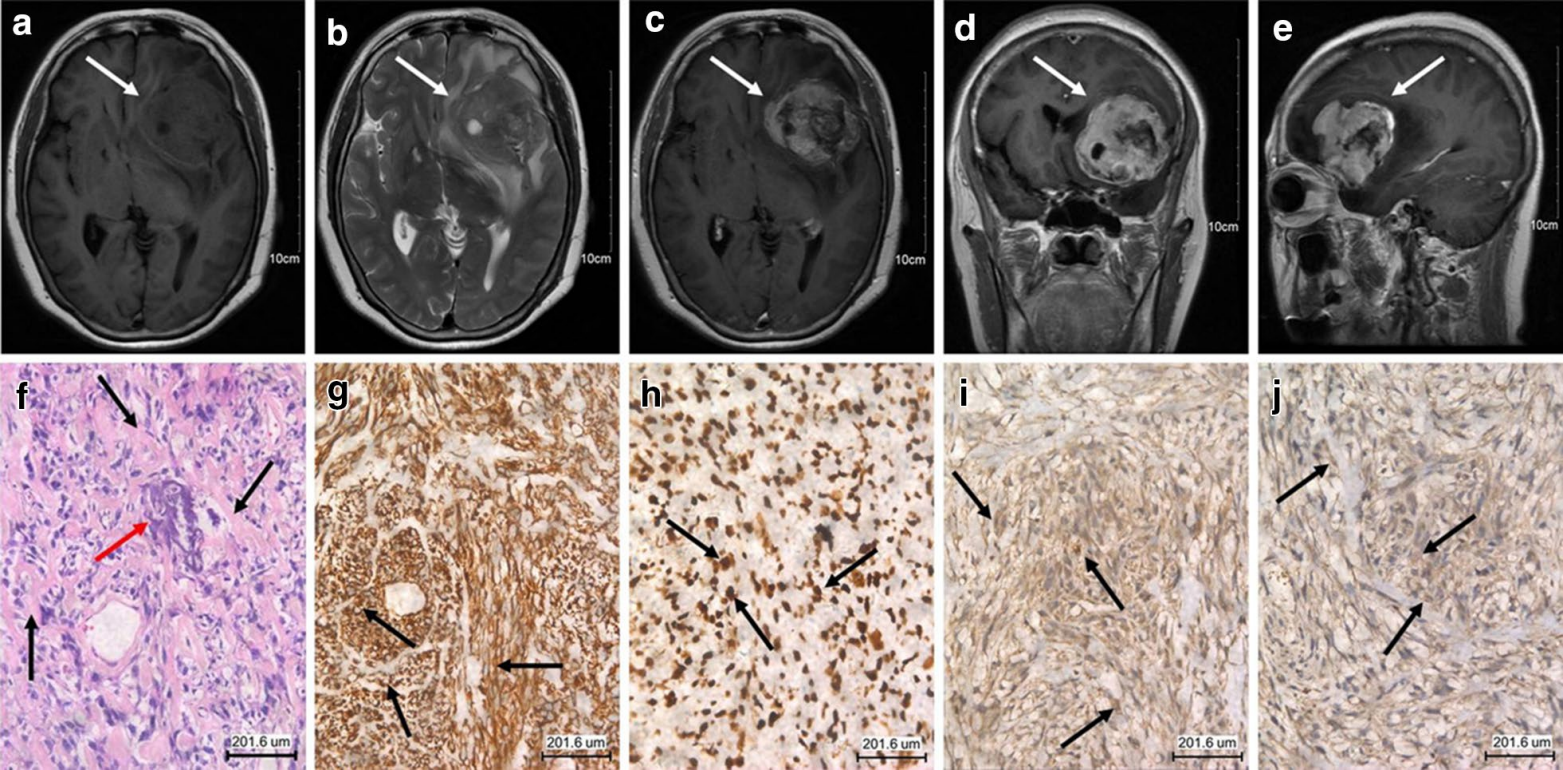
**a, b** MRI brain show a large, necrotic and calcified tumor (*white arrow*) in the parenchyma of the *left frontal lobe* with marked surrounding edema and mass effect on midline structures. The tumor appeared hypointense on non-contrast T1-weighted images and iso-to hyperintense on T2-weighted images.** c**–**e** The enhancement of the tumor was irregular and most prominent at its periphery. **f** Histopathology shows the tumor contained many neoplastic osteoid production (*black arrow*) and some calcifications (*red arrow*) (hematoxylin and eosin, original magnification, ×200), and tumor cells strongly expressing Vimentin (**g**), P53 (**h**),osteopontin (**i**) and osteonectin (**j**). (immunohistochemistry, original magnification, ×200, *black arrow*)

A 54-year-old female was referred to our institution with a 1-week history of headaches. Her past medical history was unremarkable. Magnetic resonance imaging (MRI) revealed a large, partially necrotic and calcified tumor in the parenchyma of the left frontal lobe with marked surrounding edema and mass effect on midline structures, but without any dural attachment. The tumor appeared hypointense on non-contrast T1-weighted images (Fig. 1a) and iso-to hyperintense on T2-weighted images (Fig. 1b). The enhancement of the tumor was irregular and most prominent at its periphery (Fig. 1c–e). The preoperative clinical diagnosis was meningioma or calcified glial tumor. She underwent incomplete resection of the tumor and histopathologic examination revealed a malignant mesenchymal neoplasm displaying a poorly differentiated spindle cells with interspersed eosinophilic osteoid production, calcification intimately associated with the malignant cells, and localized new bone formation (Fig. 1f). Immunohistochemistry was negative for epithelial membrane antigen (EMA), glial fibrillary acidic protein (GFAP), CD34, desmin and neurone specific enolase (NSE) but positive for vimentin (Fig. 1g), P53 (Fig. 1h), osteopontin (Fig. 1i) and osteonectin (Fig. 1j). Antisera against the proliferation marker Ki-67 revealed very variable immunoreactions (80%). Given the microscopic appearance a histopathologic diagnosis of primary intracerebral osteosarcoma was made.

Extraskeletal osteosarcoma is rare and is defined as a malignant mesenchymal neoplasm that produces osteoid as well as bone or chondroid material and is located in the soft tissue without any bony attachment (Chung and Enzinger 1987). Primary intracranial osteosarcoma most often represent intracranial invasion from a tumor arising from the skull (Salvati et al. 1993), and meningeal osteosarcomas which arise from the mesenchymal components of the meninges are also reported (Dagcinar et al. 2008). However, primary intracerebral osteosarcoma is rare with very few reported cases in literature.

## Literature search

We performed a PubMed search for all cases of primary intracerebral osteosarcoma up to September 2016. Cases were analyzed for basic demographic features including age, sex, chief complaint, location, treatment, and clinical outcome (Table 1).

**Table 1:** Summary of previously reported cases of primary intracerebral osteosarcoma

Study	Age/gender	Chief complaint	Location	Treatment	Clinical outcome
Jacques et al. (1976)	53/male	Headaches and left arm weakness	Right temporo-parietal lobe	Surgery, radiotherapy	Dead at 5 months
Reznik and Lenelle (1991)	64/male	Headaches and left side hemiparesis	Right thalamus	Surgery	Dead at 3 days
Ohara et al. (1994)	57/female	Headaches	Right parietal lobe	Surgery, radiotherapy, chemotherapy	Dead at 1 year
Sipos et al. (1997)	16/female	Headaches and impaired vision	Left parietal lobe	Surgery, radiotherapy, chemotherapy	Alive at 4 years
Bauman et al. (1997)	3/female	Seizure	Right temporal lobe	Surgery, radiotherapy, chemotherapy	Alive at 18 months
Hettmer et al. (2002)	78/male	Right side weakness	Left frontal lobe	Surgery	Recurrence at 4 months

## Conclusion

Primary intracerebral osteosarcoma is an extremely rare tumor and its radiological appearance is not pathognomonic. Despite their rarity, primary intracerebral osteosarcomas should be included in the differential diagnosis, especially for other tumors such as meningioma and calcified glial tumor, and histopathology is the mainstay in establishing the diagnosis.

## Abbreviations

MRI: magnetic resonance imaging
EMA: epithelial membrane antigen
GFAP: glial fibrillary acidic protein
NSE: neurone specific enolase
